# C-Axis Oriented LiNbO_3_ Thin Film Grown by Chemical Beam Epitaxy for Surface Acoustic Wave Device Applications

**DOI:** 10.3390/s26092858

**Published:** 2026-05-02

**Authors:** Nikolay Smagin, Thanh Ngoc Kim Bui, Zakariae Oumekloul, Rahma Moalla, William Maudez, Estelle Wagner, Marc Duquennoy, Rayen Kalai Mathlouthi, Yves Deblock, Hatem Dahmani, Denis Remiens, Julien Carlier, Giacomo Benvenuti

**Affiliations:** 1 Institut d’Électronique de Microélectronique et de Nanotechnologie (IEMN), UMR CNRS 8520, Université Polytechnique Hauts-de-France, CNRS, Université de Lille, 59313 Valenciennes, France; 2 3D-Oxides, 41 Rue Henri Fabre, 01630 Saint-Genis-Pouilly, France; 3 Institut des Nanotechnologies de Lyon (INL), Université de Lyon, Ecole Centrale de Lyon, CNRS, UMR5270, 69134 Ecully, France

**Keywords:** chemical beam epitaxy, SAW, lithium niobate, thin film, electromechanical coupling

## Abstract

**Highlights:**

**What are the main findings?**

- Epitaxial LiNbO_3_ thin films were grown on C-plane sapphire using Chemical Beam Epitaxy (CBE) at 400 °C.
- SAW delay lines and resonators operating between 1 and 3 GHz were successfully demonstrated.
- Electromechanical coupling factors (*k*_t_^2^) of ~0.3% (Rayleigh) and ~3% (Sezawa) were achieved.
- COM and BVD modeling were used to extract intrinsic and effective coupling parameters.

**What are the implications of the main findings?**

- CBE provides a scalable route for the fabrication of LiNbO_3_ thin films compatible with large wafer diameters.
- Although the current coupling remains moderate, the results demonstrate the feasibility of SAW devices on CBE-grown films.
- Optimization of film thickness and resonator design could significantly improve device performance for RF filtering and passive wireless sensing.

**Abstract:**

High-frequency surface acoustic wave (SAW) devices require piezoelectric thin films combining strong electromechanical coupling, high acoustic velocity, and compatibility with scalable fabrication. Lithium niobate (LiNbO_3_) is a promising material, but the growth of high-quality thin films remains challenging because of lithium volatility and process-control issues. In this work, chemical beam epitaxy (CBE) was investigated as an alternative route for the deposition of c-axis-oriented LiNbO_3_ thin films on C-plane sapphire at a relatively low growth temperature of 400 °C. Structural characterization confirmed high crystalline quality, with clear (006) and (0012) XRD reflections and a rocking-curve full width at half maximum of 0.04°. To evaluate acoustic performance, a SAW delay line and a one-port resonator were fabricated on 350 nm thick films using e-beam lithography. The devices operated in the 1–3 GHz range and exhibited electromechanical coupling factors of about 0.3% for the Rayleigh mode at 1.7 GHz and 3% for the Sezawa mode at 2.75 GHz. Propagation velocities ranged from 5094 to 8250 m/s, and the Rayleigh-mode resonator quality factor reached about 500. These results demonstrate the feasibility of CBE-grown LiNbO_3_ films for SAW device applications.

## 1. Introduction

Surface acoustic wave (SAW) technology plays an important role in both modern radio-frequency (RF) front-end components and passive wireless sensing systems. The increasing demand for high-performance acoustic devices has stimulated the development of new piezoelectric materials and device architectures. In particular, fifth-generation (5G) New Radio applications require acoustic filters operating above 3 GHz with ultra-wide bandwidths, such as bands n77 (3.3–4.2 GHz) and n79 (4.4–5.0 GHz) [1,2]. Meeting these requirements calls for piezoelectric platforms combining high operating frequencies, large electromechanical coupling factors, and high quality factors. Similar material requirements are also relevant for passive wireless SAW sensors, which are attractive for harsh-environment monitoring because of their small size, ruggedness, battery-free operation, and compatibility with wireless interrogation [3,4,5]. To address these challenges, advanced acoustic device architectures such as IHP-SAW, FBAR, SMR, and XBAR have been developed, often relying on multilayer or suspended configurations to enhance coupling and frequency performance [6,7,8,9].

Among the candidate materials, lithium niobate (LiNbO_3_) is particularly attractive because of its strong piezoelectric response, high Curie temperature, and favorable dielectric properties [10,11]. It is therefore widely used in photonics and electronics, including optical waveguides [12], electro-optic modulators [13], SAW filters [14], and SAW sensors [15]. In recent years, thin-film LiNbO_3_ has received increasing attention because it enables higher operating frequencies, improved integration, and enhanced control of guided acoustic-wave propagation. At present, high-quality LiNbO_3_ thin films are commonly obtained by crystal-ion-slicing or SmartCut-type transfer approaches [16], as well as by wafer bonding followed by thinning and polishing [17,18]. Although these techniques preserve many of the desirable properties of bulk single crystals, they involve complex processing, relatively high cost, and limitations in thickness scaling.

In this context, chemical beam epitaxy (CBE) is of interest as an alternative route for the growth of LiNbO_3_ thin films. CBE combines aspects of molecular beam epitaxy (MBE) and chemical vapor deposition (CVD), while offering the possibility of controlling film composition and thickness together with compatibility with scalable deposition architectures [19,20]. Previous studies have already reported the optimization of the CBE process and the structural characterization of LiNbO_3_ thin films grown by this approach [21,22]. In the present work, we investigate CBE-grown LiNbO_3_ films as a platform for SAW devices. To the best of our knowledge, this study represents one of the first demonstrations of SAW operation on LiNbO_3_ thin films grown by CBE. A preliminary version of the experimental results was presented earlier in [23].

Here, LiNbO_3_ thin films were deposited on C-plane sapphire substrates [24] at a relatively low substrate temperature of 400 °C. Their structural properties were investigated by X-ray diffraction (XRD) and scanning electron microscopy (SEM). To evaluate acoustic performance, a SAW delay line and a one-port resonator were fabricated on the deposited films. Their electrical characterization allowed the identification of Rayleigh and Sezawa propagation modes and the extraction of key parameters such as electromechanical coupling, phase velocity, temperature coefficient of frequency, and quality factor. The results demonstrate the feasibility of using CBE-grown LiNbO_3_ thin films for SAW device applications, while also highlighting the need for further optimization of both film properties and device design to approach the performance levels required for advanced RF filtering and wireless sensing applications.

## 2. Materials and Methods

### 2.1. CBE Film Deposition

**Figure 1:**
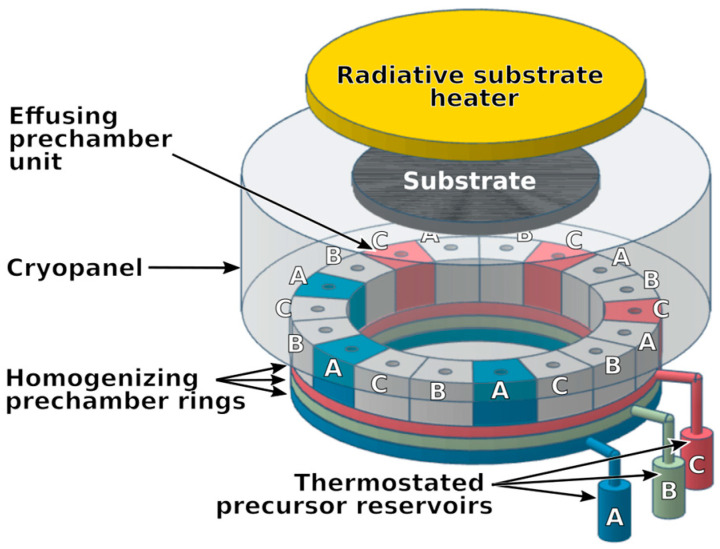
Schematic layout of the CBE system (Sybilla 200 from ABCD Technology), adapted from [21,26].

The Chemical Beam Epitaxy (CBE) [19,25] technique relies on directing volatile precursors under molecular vacuum conditions, which prevents gas-phase collisions and enables a line-of-sight deposition process [25]. In this study, a LiNbO_3_ thin film was grown on C-plane sapphire using a Sybilla 200 system (ABCD Technology, Nyon, Switzerland) (Figure 1). In this growth configuration, the crystallographic orientation of the film is governed by the substrate orientation together with the deposition conditions, particularly the substrate temperature and the Li/Nb precursor flux ratio, which favor preferential c-axis-oriented LiNbO_3_ growth.

Li precursor is located on line A, and Nb is located on line C. A temperature-controlled reservoir is used to vaporize each precursor into a pre-chamber ring, which is linked to six compartments containing effusion holes at the top. From these Knudsen apertures, the precursor molecules travel in straight paths toward the substrate, where they decompose as a result of substrate heating.

Lithium tert-butoxide ([Li(OtBu)]_6_, CAS 1907-33-1) served as the lithium precursor and was introduced via line A, while niobium tetraethoxy dimethylaminoethoxide (Nb(OEt)_4_dmae, CAS 359847-15-7) was used as the niobium precursor and supplied through line C (Figure 1). Both precursors were evaporated from heated reservoirs into a pre-chamber and subsequently directed as collimated molecular beams into the main chamber through Knudsen holes—three per source, each with a diameter of 1.5 mm. Line B, although shown in the generic Sybilla reactor scheme in Figure 1, was not used for the present LiNbO_3_ deposition.

Precursor fluxes were estimated theoretically from pre-chamber vapor pressures, which were regulated by tuning the reservoir temperatures, as described previously [21]. Under optimized conditions, the Li/Nb precursor flow ratio was calculated to be 1.44, corresponding to vapor pressures of 6.7 × 10^−3^ mbar for Li and 29.0 × 10^−3^ mbar for Nb. These pressures were achieved by setting the reservoir temperatures to 89 °C for Li and 70 °C for Nb. The substrate temperature, monitored via two thermocouples on the substrate holder, was determined to be 400 °C, driven by a 660 °C graphite heating plate. The deposition process lasted 3 h, resulting in a film thickness of approximately 350 nm.

It should be noted that, unlike in many oxygen-assisted CVD and metal-organic chemical vapor deposition (MOCVD) processes, no oxygen carrier gas is used in the present CBE configuration. As a result, the growth of significantly thicker LiNbO_3_ layers is mainly limited not by gas-phase oxygen transport, but by the difficulty of preserving stoichiometry and crystalline quality while preventing stress relaxation, twinning, and cracking as thickness increases.

### 2.2. SAW Devices Fabrication

**Figure 2:**
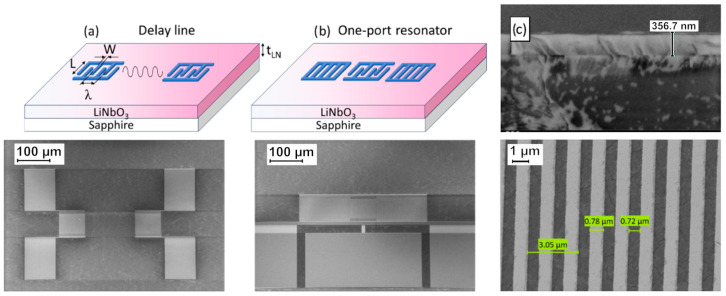
(**a**) SAW delay line (schematic (top) and SEM image (bottom)); (**b**) One-port resonator (schematic (top) and SEM image (bottom)), and (**c**) SEM image showing cross-section of LiNbO_3_ on sapphire C-plane (top) and electrode width and wavelength of IDTs (bottom).

To characterize the as-grown LiNbO_3_ film, standard electroacoustical devices—a delay line (Figure 2a) and a synchronous one-port resonator (Figure 2b)—were patterned by electron-beam lithography using a Raith EBPG 5000 Plus system (Raith B.V., Best, The Netherlands). A bi-layer PMMA photoresist was employed, with dosages of 600 µC/cm^2^ for the pads and 400 µC/cm^2^ for the fingers. The device configurations are detailed in Table 1.

Interdigital transducers (IDTs) were fabricated from aluminum, with an electrode thickness of 50 nm. The wavelength was intentionally chosen as 3 μm in order to remain within a robust lithographic process window for this first device demonstration. For the available film thickness of about 350 nm, this choice provided a compromise between manufacturability and a sufficiently large *h*_layer_/λ ratio to observe guided-wave behavior and measurable electromechanical coupling, while avoiding the use of thicker films more prone to stress relaxation and cracking on sapphire [27]. Consequently, the configuration outlined in Table 1 resulted in layer thickness-to-wavelength *h*_layer_/λ and electrode thickness-to-wavelength *h*_elec_/λ ratios of 11.7% and 1.7%, respectively. The SAW propagation direction, defined as normal to the IDT fingers, was oriented at 60° with respect to the reference flat of the sapphire substrate (i.e., the fingers themselves were at 30° to the flat). A SEM image, shown in Figure 2c, reveals a slight overdevelopment of the electrodes, leading to a width of 0.78 µm instead of the intended 0.75 µm. This corresponds to a metallization ratio of 52%, compared to the target value of 50%.

The electrical response of the SAW devices was measured using a manual radio frequency (RF) probe station connected to a Rohde and Schwarz (Rohde & Schwarz GmbH & Co. KG, Munich, Germany) ZNB Vector Network Analyzer (VNA). For characterization, RF probes with a 500 µm pitch were used in two configurations: signal–ground (S–G) and ground–signal–ground (G–S–G). The probes were supplied by T+ Co., Ltd. (Shiroi, Chiba, Japan). The TCF was measured using the VNA and an MPS-PT Peltier heating and cooling stage by Nextron (Nextron Corporation, Busan, Republic of Korea).

### 2.3. Electromechanical Coupling Factor Estimation

The electromechanical coupling factor *k*_t_^2^ was originally defined for thickness-mode resonators as [28]:

*k*_t_^2^∙= (π/2∙*f*_r_/*f*_a_)/tan(π/2∙*f*_r_/*f*_a_), (1)

where *f*_r_ is the resonance frequency, taken at the maximum conductance Re(*Y*_11_), and *f*_a_ is the anti-resonance frequency, taken at the maximum resistance Re(1/*Y*_11_), for a single-mode resonator [29]. *k*_t_^2^ is commonly used to describe the electromechanical coupling of the propagating SAW mode.

The device-level effective coupling coefficient *k*^2^_eff_ is also derived for thickness plate modes in IEEE Std. 176 [29]:

*k*^2^*_eff_* = (π/2)·(*f*_r_/*f*_a_)·tan(π/2∙(*f*_a_ − *f*_r_)/*f*_a_) = (*f*_a_^2^ − *f*_r_^2^)/*f*_a_^2^. (2)

Since this expression is derived for thickness plate modes, its application to SAW resonators should be regarded as an approximation.

Another material-level quantity is the piezoelectric coupling factor *K*^2^, a dimensionless parameter for describing a material’s capability of energy conversion between the electrical and mechanical domains:

*K*^2^ = (*f*_a_^2^ − *f*_r_^2^)/*f*_r_^2^ = *k*^2^_eff_/(1 − *k*^2^_eff_). (3)

For low to moderate coupling, *k*_t_^2^ and *K*^2^ are approximately related by [28]:

*k*_t_^2^ = π^2^/8·(*f*_a_^2^ − *f*_r_^2^)/*f*_r_^2^ = π^2^/8·*K*^2^. (4)

The resonator figure of merit can then be expressed as [28,29]:

FoM = *Q*∙*K*^2^ = *Q*∙*k*^2^_eff_/(1 − *k*^2^_eff_) ≈ (8/π^2^)∙*k*_t_^2^∙*Q*, (5)

where *Q* is the quality factor, calculated as *Q* = *f*_r_/Δ*f*_FWHM_, and Δ*f*_FWHM_ is the full frequency width at half maximum of the conductance peak [30].

## 3. Results

### 3.1. Orientation and Quality of LiNbO_3_ Film

Achieving high-quality piezoelectric films with physical characteristics approaching those of single crystals remains challenging, requiring careful control over several factors, including the layer’s composition and stoichiometry (notably Li content and the elimination of secondary phases) [31], well-defined in-plane and out-of-plane crystallographic orientations, ferroelectric domain configuration, and surface smoothness.

Raman spectroscopy provides detailed phase information for LiNbO_3_ and can also be used to quantify its lithium stoichiometry. In particular, the molar percentage of lithium (mol % Li) is linearly related to the full width at half maximum (FWHM) of two characteristic Raman peaks: the E(TO) mode at 152 cm^−1^ and the A_1_(LO) mode at 870 cm^−1^ [26]. Both modes gave a consistent lithium content of approximately 48.7–48.9 mol %. The surface topography of the fabricated film has been scanned with an atomic force microscope within the 1 × 1 μm^2^ area, giving a low root mean square value equal to 2.0 nm [26].

**Figure 3:**
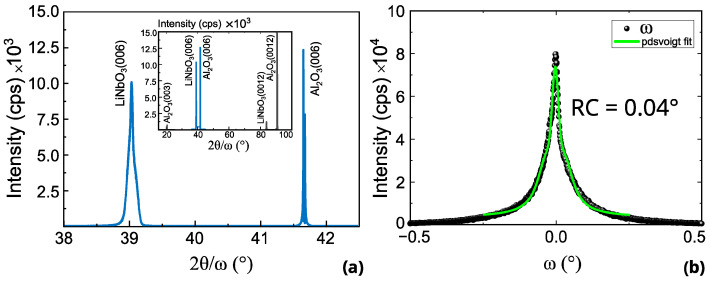
(**a**) High-resolution X-ray diffractogram 2θ/ω scan of LiNbO_3_ from 36° to 42.2° zooming in (006) plane, the full diagram in the 15–100° range is shown in the inset; (**b**) Mosaicity obtained by ω-scan on (006) plane, adapted from [26].

The crystal structure of LiNbO_3_ films was analyzed by high-resolution XRD. The 2θ/ω pattern shown in Figure 3a and the rocking curve scan of LiNbO_3_ (006) shown in Figure 3b indicate the formation of a single-phase LiNbO_3_ film, with no observable diffraction signals attributable to secondary phases such as Li_3_NbO_4_ or LiNb_3_O_8_. The film exhibits a single orientation along the C-axis, as evidenced by diffraction peaks at 38.95° and 83.68°, corresponding to the (006) and (0012) planes, respectively. To evaluate crystallite quality, rocking curve measurements were performed on the (006) reflection. The FWHM of rocking curve scans on the LiNbO_3_ (006) peak was found to be 0.04° (see Figure 3b). This value aligns with earlier reported results [21], and is comparable to those obtained for LiNbO_3_ thin films fabricated using the SmartCut process [32] (0.12°) [33]. Nonetheless, the realization of bulk acoustic wave (BAW) resonators on CVD-deposited LiNbO_3_ films [34]—with a FWHM of rocking curve of 0.4° and an electromechanical coupling factor (*k*_t_^2^) of 5.8% at 2.9 GHz—demonstrates the feasibility of alternative deposition approaches for acoustic applications.

To position the structural quality of the present CBE-grown LiNbO_3_33_ films with respect to representative directly grown LiNbO_3_ thin films relevant to acoustic applications, a comparison is provided in Table 2.

### 3.2. Characteristics of the Delay Line

**Figure 4:**
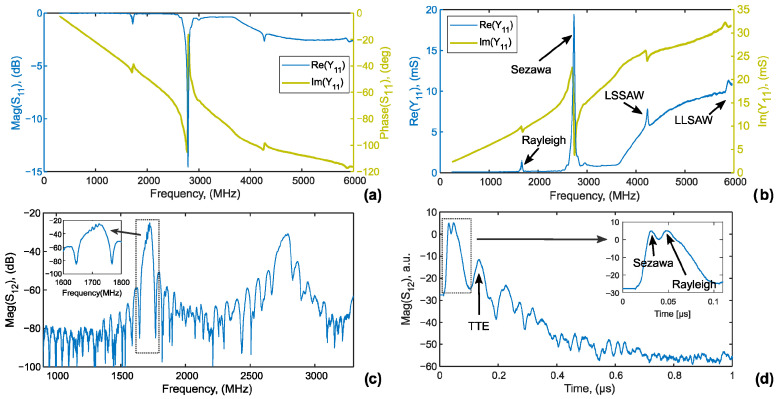
Electrical characterization of the delay line: (**a**) magnitude and phase of *S*_11_ parameter; (**b**) conductance and susceptance; (**c**) magnitude of insertion loss; (**d**) impulse response.

For a delay line, both the reflection *S*_11_ (Figure 4a) and transmission *S*_12_ (Figure 4c) scattering parameters were measured. The raw VNA measurement data presented in this paper are provided as Supplementary Materials in .s2p file format. The VNA data presented in Figure 4 were de-embedded from a 12.5 Ω series resistance in order to remove the contribution of the measurement setup and assess the intrinsic device response. Return loss *S*_11_ relates to the efficiency of electrical-to-mechanical energy conversion and is closely related to the electro-mechanical coupling factor *k*_t_^2^. Low *S*_11_ value across the operating frequency band indicates minimal power reflection, signifying good impedance matching between the transducer and the external circuitry. The *S*_11_ and *Y*_11_ curves, shown in Figure 4a,b, respectively, highlight two primary responses corresponding to the Rayleigh mode (at 1.7 GHz) and the Sezawa mode (at 2.75 GHz). It is worth mentioning that SAW devices utilizing Sezawa waves are attractive for filtering applications and for use in electroacoustic and sensing technologies [38]. Moreover, weak responses from leaky shear (LSSAW) and longitudinal (LLSAW) modes are observed at 4.4 and 5.9 GHz, respectively [35,39].

The return loss values shown in Figure 4a are −0.8 dB for the Rayleigh mode and −14.5 dB for the Sezawa mode. Admittance data presented in Figure 4b allow estimating the *k*_t_^2^ value using the following equation [40]:

*k*_t_^2^ = *G*_0_/(4π∙η^2^*f*_0_*C*_T_*N*_i_), (6)

where *C*_T_ = *W*(ε_0_ + ε_p_)*N*_i_ is static capacitance of the IDT, ε_p_ is effective permittivity of the substrate, ε_0_ = 8.854187 × 10^−12^ F × m^−1^ is permittivity of the free space, *f*_0_ is the center frequency of the device, *G*_0_ = Re(*Y*_11_) is conductance at *f*_0_, *N*_i_ is the number of IDT finger pairs, *W* is the transducer aperture, η is the IDT element factor, equal to 0.8472 for 50% metallization ratio. The static capacitance *C*_T_ can be extracted from the admittance measurements using the following equation [39]:

*C*_T_ = *B*_0_/(2π·*f*_0_), (7)

where *B*_0_ = Im(*Y*_11_) is susceptance at *f*_0_. Using Equations (6) and (7), the estimated *k*_t_^2^ value is 0.34% for the Rayleigh and 2.8% for the Sezawa mode.

Insertion loss levels of −25.2 dB for Rayleigh and −31.1 dB for Sezawa modes are acceptable for a generic (without explicit optimization) delay line using basic bidirectional IDTs deposited on a thin film [41,42,43]. The asymmetry observed in the bandpass lobe for the Rayleigh mode (Figure 4c), marked by the shift of the transmission maximum to the right relative to the stopband center, is attributed to the relatively high negative reflectivity of the electrodes [30]. This reflectivity also contributes to the triple-transit echo (TTE), which is apparent in the time-domain representation of the *S*_12_ (Figure 4d) and manifests as ripples in the passband observed in the frequency domain (inset in Figure 4c). Therefore, a non-negligible TTE ratio of 16.4 dB is observed for the Rayleigh mode (Figure 4d). Two overlapping echoes of Rayleigh and Sezawa pulses are clearly seen in the time domain (see the inset of Figure 4d). Due to the inherent nature of the Sezawa mode to propagate at the layer-substrate interface, the observed electrodes’ reflectivity and TTE are almost negligible compared to the Rayleigh mode.

### 3.3. Characteristics of the SAW Resonator

#### 3.3.1. Extracting Substrate Parameters

**Figure 5:**
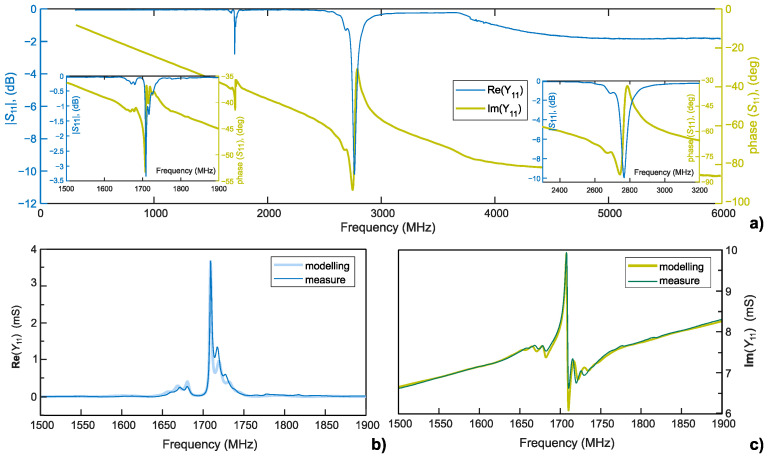
(**a**) Measured reflection coefficient *S*_11_; insets show enlarged views of the Rayleigh-mode resonance (bottom left) and Sezawa-mode resonance (bottom right). (**b**,**c**) Admittance response (conductance and susceptance, on the left and right, respectively) of the Rayleigh mode fitted with results from COM simulations.

Electrical response (*S*_11_) of the one-port resonator in the range of 1–6 GHz is presented in Figure 5a. As for the delay line, two main peaks are visible at 1.7 GHz (−2.7 dB) and 2.75 GHz (−10.1 dB) corresponding to Rayleigh and Sezawa propagation modes, respectively.

The chosen configuration of a synchronous resonator facilitates the extraction of important piezoelectric substrate parameters by fitting experimental results using the Coupling-of-Modes (COM) theory simulations [44,45]. A comprehensive presentation of the COM model and of the fitting approach can be found in numerous sources [30,44,45,46]. The curve superposition of the measurement data with the COM simulations is presented in Figure 5b,c for conductance Re(*Y*_11_) and susceptance Im(*Y*_11_), respectively.

As can be observed in Figure 5c, the imaginary part of the admittance does not cross the abscissa axis, resulting in reduced resonance characteristics. According to the COM simulation, this is due to the rather low electromechanical coupling of the thin film structure. A larger quantity of reflectors (>300 instead of the 100 used) is needed to achieve the full resonance condition, when *G*(*f*_r_) = *G*(*f*_a_) = 0.

The totality of extracted parameters is summarized in Table 3. The parameters are as follows: *f*_r_ is the resonance frequency (conductance maximum); *k*_t_^2^ is intrinsic propagating-mode coupling; *v* is mode velocity, *κ*_λ_ is reflectivity per wavelength; α_n_ is SAW attenuation; ζ_n_ is normalized transduction coefficient [44], and *C*_n_ is normalized static capacitance. The extracted *Q* values were determined from the conductance peaks using the definition introduced in Equation (5). TCF values were determined by tracking the shift of the resonance peak as the temperature of the Peltier element varied from 25 °C to 80 °C and vice versa. The measured values are −66 ppm and −58 ppm for the Rayleigh and Sezawa modes, respectively. The lower absolute TCF value of the Sezawa wave is attributed to its higher proximity to the sapphire substrate.

The third line in Table 3 (“Rayleigh-bulk”) represents the results of modeling the same resonator structure on a bulk LiNbO_3_. The COM parameters for Al electrodes on bulk LiNbO_3_ can be found in [30] (p. 220). The resonance frequency of such a structure would be 1.29 GHz, corresponding to the SAW velocity of 3870 m/s.

The inversion of the reflectivity sign κ_λ_ observed in Table 3 for the Rayleigh modes on the multilayer structure and the monocrystalline substrate is not immediately evident. This behavior may be attributed to the different balance between mass loading and electrical loading introduced by the electrodes. Since this balance depends on the substrate properties and the acoustic field distribution, variations in the relative contributions of these mechanisms can modify the phase of the acoustic reflection at the electrode. As a result, the effective reflectivity coefficient may change sign.

It is worth noting that the *k*_t_^2^ values are consistent with those extracted for the delay line using Equations (6) and (7). COM analysis relates the acoustic radiation strength of the IDT to the transduction coefficient ζ, which is proportional to the electromechanical coupling factor *k*_t_^2^ of the propagating SAW mode. The coupling obtained from COM therefore corresponds to the periodic transduction of a propagating SAW under a uniform IDT, i.e., the limit of an infinitely long transducer. In Equation (6), normalization by the number of finger pairs *N*_i_ removes the finite-transducer dependence of the IDT admittance and isolates the transduction strength per period. Conceptually, both approaches therefore correspond to the same propagating SAW-mode coupling coefficient.

#### 3.3.2. Extracting SAW Resonators’ Effective Parameters

While the coupling values obtained from COM analysis and delay-line extraction describe the intrinsic electromechanical coupling of the propagating SAW mode, they represent the coupling strength in the limit of a periodic transducer (infinitely long IDT [30]) and therefore provide an upper bound for the achievable interaction in a resonator. In a practical device, the finite acoustic cavity, reflector efficiency, and static capacitance modify the resonance–antiresonance separation observed in the electrical response.

**Figure 6:**
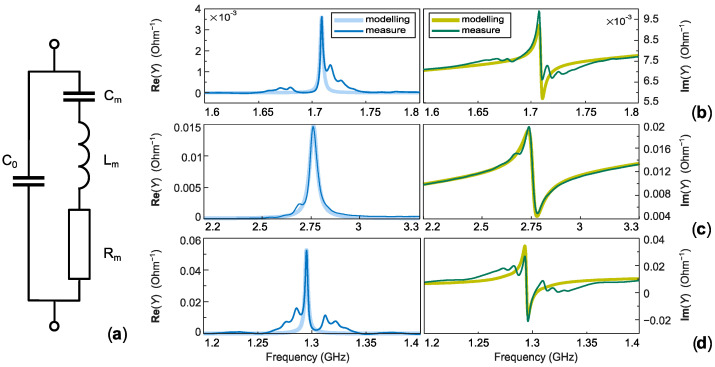
(**a**) BVD equivalent circuit. (**b**–**d**) Admittance response (conductance and susceptance, on the left and right, respectively) fitted BVD equivalent circuit approximation for the Rayleigh, the Sezawa, and the Rayleigh mode simulated for bulk LiNbO_3_, respectively.

Consequently, the effective coupling coefficients *k*^2^_eff_ and *k^2^*_t,BVD_ are introduced in the following to characterize the performance of the fabricated resonator. The effective coupling coefficient *k*^2^_eff_ is estimated using Equation (2), while the effective electromechanical coupling factor *k^2^*_t,BVD_ can be estimated using the well-known Butterworth–Van Dyke (BVD) equivalent circuit, shown in Figure 6a.

The BVD model represents the electromechanical response of the resonator using a static capacitance *C*_0_ in parallel with a motional branch composed of *R*_m_, *L*_m_ and *C*_m_, corresponding respectively to acoustic losses, inertia, and elastic energy storage of the resonant mode. Within this representation, the effective electromechanical coupling can be expressed as the ratio of the motional to static capacitance [28,30]:

*k*^2^*_t_*_,*BVD*_ = π^2^/8∙*C*_m_/*C*_0_. (8)

The parameters of the motional branch were extracted from the measured admittance using the relations *R*_m_ = 1/*G*_0_, *C*_m_ = 1/ω_r_*R*_m_*Q*, *L*_m_ = 1/ω_r_^2^*C*_m_, where ω_r_ = 2π*f*_r_, and the static capacitance *C*_0_ was obtained from a linear fit of the susceptance curve outside the resonance region [29]. The measured admittance responses together with the corresponding BVD fits are shown in Figure 6a–c for the Rayleigh mode, the Sezawa mode, and the Rayleigh mode simulated for bulk LiNbO_3_, respectively.

It should be noted that the anti-resonance frequency cannot be accurately reproduced using a single-branch BVD model due to the presence of secondary weak resonances originating from ripples in the reflection coefficient of the side gratings surrounding the acoustic cavity. These ripples introduce additional resonant features in the admittance response that are not captured by the simplified equivalent circuit. Nevertheless, as can be observed in Figure 6, the resonance frequency *f*_r_ and the quality factor *Q* of the dominant mode are well preserved by the model.

A more accurate representation of the Rayleigh-mode response would require the introduction of additional parallel motional branches in the equivalent circuit to account for these secondary resonances [28]. Such an approach would increase the model complexity but is not expected to significantly affect the parameters associated with the dominant resonance. Therefore, the single-branch BVD model was considered sufficient for estimating the effective coupling coefficient of the primary mode.

Table 4 summarizes the BVD equivalent circuit parameters extracted from the admittance measurements together with the derived effective parameters of the investigated modes. A good consistency can be observed between the coupling coefficients *k*^2^_eff_ obtained from the resonance–antiresonance separation and *k*^2^_*t*,*BVD*_ estimated from the ratio *C*_m_/*C*_0_. This agreement confirms that both estimators describe the same electromechanical interaction of the dominant resonant mode within the single-resonance approximation. The figure of merit values reported in Table 4 were calculated based on *k*^2^_*t*,*BVD*_, according to Equation (5).

As expected, the effective electromechanical coupling factors extracted from the resonator response are lower than those obtained for the propagating SAW modes. In particular, the coupling coefficient is reduced to approximately 0.11% for the Rayleigh mode (denoted as “R” in Table 4) compared to 0.3% obtained from the SAW propagation analysis. Similarly, the Sezawa mode (denoted as “S” in Table 4) exhibits an effective coupling of about 2.5%, whereas the coupling extracted for the propagating mode is approximately 3%.

This difference is expected, since the effective coupling factors derived from the resonator response are device-dependent and are influenced by the cavity configuration, reflector efficiency, and the static capacitance of the transducer. Consequently, the resonator geometry must be carefully optimized in order to maximize the achievable figure of merit for a given substrate.

For comparison, the simulated resonator implemented on bulk LiNbO_3_ (“R-bulk” in Table 4) exhibits a significantly higher figure of merit than the two previously considered configurations. However, the obtained FoM remains relatively low (below 10) in absolute terms [29]. This behavior can be attributed to the fact that the resonator mask was not specifically optimized for this substrate, which limits the achievable effective coupling and quality factor. Nevertheless, it should be noted that FoM values in the range of 0.8–2 already indicate sufficient performance for wireless interrogation in sensing applications [48,49].

## 4. Discussion

This study reports the characterization of CBE-grown LiNbO_3_ thin films for potential SAW device applications. The films, with a thickness of approximately 350 nm, were epitaxially deposited on C-plane sapphire substrates, and structural characterization confirmed high crystalline quality and the expected crystallographic orientation. XRD measurements revealed clear (006) and (0012) reflections with a rocking-curve FWHM of 0.04°, indicating a well-oriented epitaxial film.

It should be emphasized that the present study does not include a wafer-scale mapping of LiNbO_3_ thickness or composition uniformity. Nevertheless, previous reactor-development studies on the Sybilla CBVD platform demonstrated homogeneous deposition over large substrates, with thickness non-uniformity of about 5% on 450 mm wafers [19] and about 3% on 150 mm wafers. Mechanical properties such as scratch resistance and quantitative adhesion to the sapphire substrate were not specifically investigated in the present study, which is focused on the acoustic feasibility of the LiNbO_3_ sapphire system.

SAW devices fabricated on these films exhibited electromechanical coupling factors of approximately 0.3% for the Rayleigh mode at 1.7 GHz and about 3% for the Sezawa mode at 2.75 GHz. The measured propagation velocities ranged from 5094 m/s for the Rayleigh mode to 8250 m/s for the Sezawa mode, while the resonator quality factors were approximately 500 and 60, respectively. In addition, the delay lines demonstrated insertion losses around −25 dB, confirming the acoustic functionality of the fabricated structures. An additional simulation performed for the same resonator geometry on bulk LiNbO_3_ yielded an effective coupling of 1.7% and a FoM of 8.4, further illustrating that the resonator design must be specifically optimized for each material platform. A meaningful quantitative cost comparison with conventional bulk LiNbO_3_-based devices cannot yet be established, since the economics depend strongly on substrate specification, device architecture, and manufacturing scale.

The recent literature highlights that the performance gap between the present CBE-grown LiNbO_3_ demonstrators and state-of-the-art thin-film LiNbO_3_ acoustic devices is now driven not only by film quality, but also, and often predominantly, by device architecture and wave-mode engineering. While transferred single-crystal platforms obtained by crystal-ion-slicing/SmartCut remain the dominant route for high-performance thin-film LiNbO_3_ [50], direct-growth approaches such as MBE, PLD, MOCVD and sputtering [51] continue to be explored because of their potential for lower-cost and scalable fabrication. In parallel, the most significant recent advances in SAW performance have been achieved through engineered multilayer platforms rather than through further optimization of conventional resonator designs alone. For instance, a 41°YX LiNbO_3_ thin-film SAW resonator prepared by crystal-ion-slicing and bonding reported an effective electromechanical coupling of 33.54% with *Q* ≈ 380 (FoM ≈ 127) in 2023 [32], while more recent multilayer LiNbO_3_ platforms have demonstrated *k*^2^_t_ values of 7.6–8.9% with *Q* up to 679 (FoM ≈ 54) [52], as well as LiNbO_3_-on-SiC resonators and filters with *k*^2^_t_ in the 14–28% range and FoM from 166 to 222 at 5–6 GHz [53]. These comparisons indicate that the moderate coupling values obtained here (~0.3% for the Rayleigh mode and ~3% for the Sezawa mode) should not be interpreted solely as a limitation of the CBE-grown film itself. In this context, the present work should be viewed as a first proof-of-feasibility for SAW operation on CBE-grown LiNbO_3_ thin films, providing a basis for future optimization of both the film stack and the device design.

The limitation of electromechanical coupling factors is, in particular, related to the relatively small thickness-to-wavelength ratio used in this work. Previous studies have shown that the electromechanical coupling in layered structures is maximized when the ratio *h*_layer_/λ approaches 40–50% [30,35]. Achieving this condition would require the fabrication of LiNbO_3_ layers with thicknesses approaching 1.5 µm, which remains challenging due to the relaxation of residual stresses through twin formation and cracking [27,43].

Alternatively, reducing the acoustic wavelength while maintaining the current film thickness could improve the effective coupling by increasing the *h*_layer_/λ ratio. Future work will therefore investigate thinner films (≈200 nm) and optimized device geometries operating at higher frequencies in order to enhance the electromechanical coupling. Improvements in crystalline quality and defect reduction are also expected to further enhance device performance. Finally, the investigated films may also enable the implementation of BAW-type devices through the integration of bottom electrodes.

## 5. Conclusions

This work demonstrates the feasibility of CBE for the growth of LiNbO_3_33_ thin films suitable for SAW device fabrication. High-quality epitaxial films were obtained on C-plane sapphire substrates, as confirmed by XRD measurements showing clear (006) and (0012) reflections and a rocking-curve FWHM of 0.04°. A SAW delay line and one-port resonator fabricated on these films enabled the identification of Rayleigh and Sezawa modes, with measured propagation velocities of 5094 m/s and 8250 m/s, respectively, and electromechanical coupling factors of approximately 0.3% and 3%.

Although these values remain below those reported for state-of-the-art thin-film LiNbO_3_ acoustic platforms, the present results should be interpreted as a first proof of feasibility for SAW operation on CBE-grown LiNbO_3_ films rather than as the performance limit of the material system. The moderate *k*_t_^2^ values obtained here reflect not only the current film characteristics, but also the non-optimized thickness-to-wavelength ratio and the preliminary resonator architecture used in this study. More generally, the recent literature indicates that further gains will require not only improvements in film quality, but also device architecture and wave-mode engineering.

The results nevertheless confirm the potential of CBE as a scalable alternative to transferred thin-film technologies for the realization of LiNbO_3_-based acoustic devices. Further improvements are expected through optimization of film thickness, reduction of residual stress and refinement of resonator design. In particular, increasing the *h*_layer_/λ ratio and improving device architecture should enable significantly enhanced coupling and figure of merit. It should be noted that, although the present work is limited to a device-level demonstration, previous reactor-development studies have shown that the underlying CBE deposition architecture is compatible with homogeneous growth on large substrates, with demonstrated scaling from 150 mm to 450 mm wafers [19]. This scalability, combined with the encouraging first acoustic results obtained here, makes CBE-grown LiNbO_3_ thin films a promising route toward future high-frequency SAW devices and sensors.

## Figures and Tables

**Table 1:** SAW Device parameters fabricated on LiNbO_3_ thin film on Sapphire.

Parameter	Delay Line	Resonator
Electrode material	Al	Al
Electrode thickness (nm)	50	50
Film thickness (nm)	350	350
Wavelength (λ) (µm)	3	3
IDT length (L) (µm)	78	78
IDT Pairs (*N*_i_)	26	26
Electrodes in grating, (*N*_r_)	—	100
Aperture width (*W*) (µm)	60 (20λ)	60
Gap (inter IDT or IDT-reflector) (µm)	1500 (500λ)	0.75 (λ/4)

**Table 2:** Representative directly grown LiNbO_3_ thin films relevant to acoustic applications.

Ref.	Fabrication Route	Substrate/ Stack	Film Thickness	Preferred Orientation/ Epitaxy	Rocking-Curve FWHM	Acoustic Demonstration	Key Reported Acoustic Result
This work	CBE	C-plane sapphire	350 nm	c-axis; (006)/(0012)	0.04°	SAW delay line and one-port resonator	Rayleigh: k_t_^2^ ≈ 0.3% at 1.7 GHz; Sezawa: k_t_^2^ ≈ 3% at 2.75 GHz
[35]	Pulsed-injection MOCVD	C-plane sapphire	158 ± 6 nm	c-axis oriented LN; (0001)LN ‖ (0001)sapphire, [$1\bar{1}20$] LN ‖ [$1\bar{1}20$] sapphire	0.255° (φ-scan FWHM: 0.676°)	SAW resonators	3.7–5.3 GHz; Rayleigh-wave K_s_^2^ up to 8%
[36]	DLI-CVD ^1^	Z-sapphire/c-sapphire	160 nm	c-axis oriented; single in-plane orientation	0.3°	Surface elastic waves (BLS)	Rayleigh, leaky shear, and leaky longitudinal waves observed between 10 and 30 GHz
[34]	CVD	ZnO buffer/ Pt bottom electrode/membrane-type stack	~410 nm	c-axis-oriented twinned crystalline epitaxial film	~0.4°	FBAR	2.9 GHz FBAR; k_t_^2^ = 5.8%; Q_r_ ≈ 73
[37]	Solid-source MOCVD	C-plane sapphire	~110 nm	100% c-axis oriented	0.044°	Not reported	Structural/optical benchmark; sapphire growth limited by cracking above ~150 nm

^1^ DLI-CVD, direct liquid injection chemical vapor deposition.

**Table 3:** Extracted SAW device and substrate parameters for Rayleigh and Sezawa modes.

Mode	*f*_r_ (GHz)	*k*_t_^2^ (%)	*v* (m/s)	κ_λ_ (%)	α_n_(dB/λ)	ζ_n_ (√Ω)	*C*_n_ (pF/µm)	ε_p_	TCF (ppm)	*Q*
Rayleigh	1.709	0.3	5094	−3.5	0.018	16 × 10^−5^	45 × 10^−5^	49.2	−66	526
Sezawa	2.75	3	8250	−0.21	–	66 × 10^−5^	44 × 10^−5^	49.0	−58	61
Rayleigh-bulk	1.29	6.3	3870	2	0.0035 ^1^	70 × 10^−5^	49 × 10^−5^	55.2	−75	417

^1^ taken from [47] for comparable frequency.

**Table 4:** BVD equivalent circuit parameters and derived effective parameters.

Mode	*C*_0_ (pF)	*C*_m_ (fF)	*R*_m_ (Ω)	*L*_m_ (µH)	*k* ^2^ *_t_*_,*BVD*_	*k* ^2^_eff_	*Q*	FoM
R	0.697	0.62114	283.3769	13.9587	0.11%	0.1%	526	0.47
S	0.677	13.805	68.162	0.24126	2.52%	2.22%	61	1.25
R-bulk	0.761	15.322	19.2624	0.98609	2.48%	1.7%	417	8.4

## Data Availability

The data that support the findings of this study are available from the corresponding author upon reasonable request.

## Supplementary Materials

The following supporting information can be downloaded at: https://www.mdpi.com/article/10.3390/s26092858/s1.

## Abbreviations

The following abbreviations are used in this manuscript:

BAW	Bulk Acoustic Wave
BVD	Butterworth–Van Dyke equivalent circuit
CBE	Chemical Beam Epitaxy
COM	Coupling of Modes
CMR	Contour-Mode Resonator
CVD	Chemical Vapor Deposition
FBAR	Film Bulk Acoustic Resonator
FWHM	Full Width at Half Maximum
IDT	Interdigital Transducer
IHP-SAW	Incredible High-performance SAW
LLSAW	Leaky Longitudinal SAW
LSSAW	Leaky Shear SAW
MBE	Molecular Beam Epitaxy
MOCVD	Metal-Organic Chemical Vapor Deposition
SAW	Surface Acoustic Wave
SEM	Scanning Electron Microscope
TCF	Temperature Coefficient of Frequency
TTE	Triple Transit Echo
XBAR	Laterally Excited Bulk Wave Resonator
XRD	X-Ray diffraction characterization
